# An IoT enabled system for enhanced air quality monitoring and prediction on the edge

**DOI:** 10.1007/s40747-021-00476-w

**Published:** 2021-07-29

**Authors:** Ahmed Samy Moursi, Nawal El-Fishawy, Soufiene Djahel, Marwa Ahmed Shouman

**Affiliations:** 1 Computer Science and Engineering Department, Faculty of Electronic Engineering, Menoufia University, Menouf, 32952 Menoufia Governorate Egypt; 2 Department of Computing and Mathematics, Manchester Metropolitan University, Manchester, M15 6BH UK

**Keywords:** Air pollution forecast, PM_2.5_, Machine learning, NARX architecture, Edge computing, IoT

## Abstract

Air pollution is a major issue resulting from the excessive use of conventional energy sources in developing countries and worldwide. Particulate Matter less than 2.5 µm in diameter (PM_2.5_) is the most dangerous air pollutant invading the human respiratory system and causing lung and heart diseases. Therefore, innovative air pollution forecasting methods and systems are required to reduce such risk. To that end, this paper proposes an Internet of Things (IoT) enabled system for monitoring and predicting PM_2.5_ concentration on both edge devices and the cloud. This system employs a hybrid prediction architecture using several Machine Learning (ML) algorithms hosted by Nonlinear AutoRegression with eXogenous input (NARX). It uses the past 24 h of PM_2.5_, cumulated wind speed and cumulated rain hours to predict the next hour of PM_2.5_. This system was tested on a PC to evaluate cloud prediction and a Raspberry P_*i*_ to evaluate edge devices’ prediction. Such a system is essential, responding quickly to air pollution in remote areas with low bandwidth or no internet connection. The performance of our system was assessed using Root Mean Square Error (RMSE), Normalized Root Mean Square Error (NRMSE), coefficient of determination (*R*^2^), Index of Agreement (IA), and duration in seconds. The obtained results highlighted that NARX/LSTM achieved the highest *R*^2^ and IA and the least RMSE and NRMSE, outperforming other previously proposed deep learning hybrid algorithms. In contrast, NARX/XGBRF achieved the best balance between accuracy and speed on the Raspberry P_*i*_.

## Introduction

Urbanization promises a very high standard of life at the expense of deterioration in the environment and air quality. The extensive use of fossil-fuel-powered cars and machines everywhere releases a massive amount of harmful gases and particulate matter into our air. Air is a crucial component of life on Earth for every being: a plant, an animal, or a human alike. Air pollution undermines the wellbeing and development of those living creatures directly. Lately, because of that rapid urbanization, air quality is declining quickly. There are several types of air pollutants, including carbon oxides CO_x_ (CO–CO_2_), nitrogen oxides NO_x_ (NO–NO_2_), sulphur oxides SO_x_ (SO_2_, SO_3_, SO_4_), Atmospheric Particulate Matter (PM for short) of diameters less than or equal to 10 µm (PM_10_), and PM of diameter less than or equal to 2.5 µm (PM_2.5_). Researchers focus on detecting and forecasting these contaminants, preferably in real-time [1–3].

Many countries worldwide defined their policies and standards to observe air pollution and generate alerts for their citizens [4]. However, these observations are mainly for outdoor environments, and most of the measurements are static and report average values. Nonetheless, air quality varies in real-time and may be affected by many factors [1], for instance, population density, wind speed and direction, pollutant distribution, location (indoors or outdoors), and various meteorological circumstances.

Air pollution is regarded as a blend of particles and gases—whose concentration is higher than a recommended safety level—discharged into the atmosphere [5]. The sources of pollutants can be split into two main divisions: natural and anthropogenic (human-made). Pollution of natural sources refers to natural incidents triggering destructive effects on the environment or emitting harmful substances. Examples of natural incidents are forest conflagrations and volcanic outbursts, generating lots of air pollutants, including SO_x_, NO_x,_ and CO_x_. On the other hand, numerous human-made sources exist like vehicles’ emissions and fuel combustion, which are deemed one of the leading causes of air pollution. Resultant pollutants could contain particulate matter, hydrogen, metal compounds, nitrogen, sulphur, and ozone. Atmospheric particulate matter encompasses liquid or solid granular which remains suspended in the atmosphere.

Medically speaking, diverse levels of health complications inflict human beings via PM [6]. Recent studies discovered an insinuated relationship between long exposure to air pollution, especially PM_2.5,_ and an increased chance of death due to the high risk of viral infections such as COVID-19 [7]. There is evidence that PM could be a possible carrier of SARS-Cov-2 (COVID-19) both directly as a platform for viral intermixture and indirectly by inducing a substance upon exposure to PM, helping the virus to adhere to the lungs [8]. Besides, PM_2.5_ is considered accountable for about 3.3 million early deaths per year worldwide, mainly in Asia [9]. Moreover, Egypt ranks in the 11th position—by 35,000 deaths—in the top countries with premature death cases associated with outdoor air pollution. In 2016, WHO (World Health Organization) issued a report declaring Cairo (Egypt) as the second top polluted city by PM_10_ amongst mega-cities of population surpassing 14 million residents and the highest level of PM_10_ in Low- and Middle-Income Countries (LMIC) of Eastern Mediterranean (Emr) for the interval of (2011–2015) [10]. There have been great work-in-progress efforts to lower the average PM_2.5_ level in Egypt as indicated in the change from 78.5 to 67.9 µg/m^3^ (a change of about 11 µg/m^3^) during the period from 2010 to 2019 [11]. Still, it is much higher than WHO guideline (10 µg/m^3^) and WHO least-stringent intermediate goal, Interim Target 1, (35 µg/m^3^) as well as the global average of 42.6 µg/m^3^ in 2019 [11]. In addition, mortality rate related to PM_2.5_ in Egypt is the highest in North Africa and the Middle East region having 91,000 such deaths [11].

Currently, a lot of research attention are devoted to improving air quality and air pollution control [11, 12]. Developing accurate techniques and tools to ensure air quality monitoring and prediction is crucial to achieving that goal. Predicting or forecasting is a vital part of the machine learning research field, which can deduce the future variation of an object’s state relative to previously collected data. Pollution forecasting is the projection of pollutant concentration in the short or long term. Research on air pollution control has evolved since the 1960s. This evolution led to an increased awareness of the population about the devastating effect of this issue. Therefore, this led to a shift of the research focus towards air pollution forecasting.

According to how the prediction process is performed, air pollution forecasting is split into three categories: numerical models, statistical models, and potential forecasts. Moreover, it can be categorized into only two types based on forecast: pollution potential forecasting and concentration forecasting [12].

Numerical modelling, as well as statistical methods, can be used for forecasting pollutants concentration. Nevertheless, the potential forecast can foretell the capacity and ability of meteorological factors, such as temperature and wind speed, along with other factors to dilute or diffuse air pollutants. If the weather conditions are likely to match the standards for possible severe pollution, a warning will be issued. In Egypt, potential forecasting is the primary tool to predict air quality [13]. Concentration forecast can predict pollutants concentration directly in a specific area, and the forecasted values are quantitative. Predicting air quality usually uses meteorological features besides pollutant concentrations to better predict future concentrations. However, in [14], data from various sources, including satellite images and measured data from ground stations, were combined for better prediction.

Linear machine learning (ML) models are employed in statistics and computer science to solve prediction problems in a data-driven approach, primarily when using multiple linear regression [15]. However, the air pollutant behaviour is primarily non-linear, so Support Vector Regression (SVR) could be used [16]. Nonetheless, a recent study shows that deep learning-based methods are generally more accurate in predicting air pollutants [17]. Therefore, multiple non-linear algorithms and deep learning-based algorithms were used in this paper to predict PM_2.5_ for the next hour using the data collected during the prior 24 h.

Conventionally, air quality is measured using air pollution monitoring stations abundant in sizes and expensive for installation and maintenance [18]. However, air-quality data generated by these stations are very accurate. According to Egypt’s vision of 2030, there will be an increase in stations deployed across the country up to 120 stations [19]. These stations will cost a lot. Alternative solutions have been suggested to be more cost-effective and therefore cover larger areas. Internet of Things (IoT) is a relatively new technology that attracts the interest of both academia and industry. To overcome the shortcomings of existing air pollution monitoring systems in detecting and predicting near future air pollution and reducing the overall cost, this paper introduces a novel approach that infuses IoT technology with environmental monitoring and the power of edge computing and machine learning. This approach provides a relatively low-cost, accurate reporting, predictive, easy to deploy, scalable and user-friendly system. Multiple algorithms are evaluated on a PC and a Raspberry Pi to test for accuracy and speed for centralized and edge prediction. Prediction on edge devices is crucial to respond quickly to air pollution incidents in faraway regions with weak or no connection to the internet, which is the case for many low- or medium-income countries.

Specifically, the main contributions of this paper are summarized as follows:

- Proposing a new IoT enabled and Edge computing-based system for air quality monitoring and prediction.
- Proposing and evaluating a Non-linear AutoRegression with eXogenous input (NARX) hybrid architecture using machine learning algorithms for edge prediction scenarios and central prediction.
- Testing the proposed NARX architecture on a PC for central prediction evaluation and a Raspberry Pi 4 for edge prediction.
- Evaluating many non-linear algorithms, including Long Short-Term Memory (LSTM), Random Forest, Extra Trees, Gradient Boost, Extreme Gradient Boost, and Random Forests in XGBoost using the proposed NARX architecture.
- Comparing our proposed architecture against the APNet algorithm proposed in [20] in terms of RMSE and IA, it was found that the NARX/LSTM hybrid algorithm produces better results than APNet

The remainder of this paper is structured as follows. Section “Related Work” reviews the most important relevant works in the literature, and “Proposed IoT-based air quality monitoring and prediction system description” demonstrates briefly the hybrid machine learning algorithms used in this work. Section “Proposed NARX hybrid architecture” describes the proposed hybrid NARX algorithm architecture. Section “Performance Evaluation” presents the evaluation metrics used in this study. Section “Data description and preprocessing” introduces the dataset used and describes how the preprocessing was performed. Finally, Sect. “Results analysis and discussion” analyses and discusses the study results on both the PC and Raspberry Pi 4 configurations, and Sect. “Conclusion” draws the concluding remarks and outcomes of this study.

## Related work

Predicting atmospheric particulate matter has significant importance; researchers examined methods seeking APM /PM concentrations forecast as accurate and as early as they can. However, using these methods in the real world imposed the need for systems that can use sensors to collect raw environmental readings to monitor pollution and machine learning algorithms to predict the next pollution level.

APNet has been presented by [20], combining LSTM and CNN to predict PM_2.5_ in a smart city configuration better. They used the past 24 h data of PM_2.5_ concentration along with cumulated hours of rain and cumulated windspeed to predict the next hour using the dataset in [21]. Their proposal outperformed LSTM and CNN individually as well as other machine learning algorithms. They evaluated their proposal using Mean Absolute Error (MAE), Root Mean Square Error (RMSE), Pearson correlation coefficient and Index of Agreement (IA). They verified the feasibility and practicality for forecasting PM_2.5_ using their proposal experimentally. Nonetheless, because the source of PM_2.5_ pollution is unstable, the real trend was not followed accurately by algorithm predictions and was a bit shifted and disordered.

A hybrid deep learning model was proposed by [22] that used LSTM with Convolutional Neural Network (CNN) and LSTM with Gated Recurrent Unit (GRU) to better forecast PM_2.5_ and PM_10_, respectively, for the next seven days. Their experiments were evaluated by RMSE and MAE. For five randomly selected areas, their hybrid models performed better than other single models. CNN-GRU and CNN-LSTM were better fitted for PM_10_ and PM_2.5_, respectively. However, the future highest and lowest levels of PM_2.5_ were weakly predicted by these hybrid models. Also, in [23], a comparison between four machine learning algorithms (Support Vector Regression (SVR), Long Short-Term Memory (LSTM), Random Forest, and Extra Trees) was made. They used the past 48 h to predict the next hour. The study was limited in the number of machine learning algorithms compared. There was a bit of a shift between actual and predicted values for most algorithms. It was found that the Extra Trees algorithm gives the best prediction performance in terms of RMSE, coefficient of determination *R*^2^.

Another hybrid deep learning multivariate CNN-LSTM model was developed in [24] to predict PM_2.5_ concentration for the next 24 h in Beijing using the past seven days data from the dataset introduced in [21]. CNN could extract air quality features, shortening training time where LSTM could perform prediction using long-term historical input data. They tested both univariate and multivariate versions of CNN-LSTM against LSTM only version. To evaluate their work, RMSE and MAE were used. However, more evaluation parameters, stating closeness to real values like *R*^2^ or IA rather than only errors metrics, could have been used to confirm their models’ performance.

To predict the daily averaged concentration of PM_10_ for one to three days ahead, [25] used meteorological parameters and history of PM_10_ in three setups for comparison purposes. The setups were a multiple linear regression model and a neural network model that uses recursive and non-recursive architectures. In addition, carbon monoxide was included as an input parameter and as a result brought performance enhancement to the prediction. Finally, PM_2.5_ concentration was predicted using meteorological parameters and PM_10_ and CO without a history of PM_2.5_ itself. They used correlation coefficient (*R*), Normalized Mean Squared Error (NMSE), fractional bias (FB) and Factor of 2 (FA2) as evaluation parameters. The recursive artificial neural network model was the best in all the conducted experiments. However, more machine learning models could have been used to test their methodology further.

Some of the literature tackled the lack of air quality measurement equipment in every location using Spatio-temporal algorithms. These algorithms predict air quality at a location and a time depending on another measurement elsewhere. The same technique can be used to enhance prediction at a location depending on measurements taken around it. A solution proposed in [26] used data of PM_2.5_, PM_10_ and O_3_ to predict air quality of the next 48 h using the 72-h history of features for every monitoring station in London and Beijing. They designed local and global air quality features by developing LightGBM, Gated-DNN and Seq2Seq. LightGBM was used as a feature selector, while Gated-DNN captured the temporal and spatial–temporal correlations, and Seq2Seq comprised an encoder summarizing historical features and a decoder that included predicted meteorological data as input, thus improving the accuracy. The ensemble of the three models (AccuAir) proved to be better than the individual components tested. Their models were evaluated using Symmetric Mean Absolute Percentage Error (SMAPE). They did not use LSTM in their Seq2Seq model, although it was proven to be very efficient in time series prediction.

Another study [27] used spatiotemporal correlation analysis for 384 monitoring stations across China with Beijing City at the centre to form a spatiotemporal feature vector (STFV). This vector reflected both linear and non-linear features of historical air quality and meteorological data and was formed using mutual information (MI) correlation analysis. The PM predictor was composed of CNN and LSTM to predict the next day’s PM_2.5_ average concentration. They experimented on data collected during three years and was evaluated using RMSE, MAE and Mean Absolute Percentage Error (MAPE). Their model was compared to Multilayer Perceptron (MLP) and LSTM models and proved to be more stable and accurate. However, their system predicts only the daily average and cannot be deployed to predict the hourly or real-time concentration of PM_2.5_.

As for IoT systems that monitor air quality and, in some cases, predict it, plenty of proposed systems exist. However, prediction in all of them is made in the cloud rather than at the edge. Chen Xiaojun et al. [28] suggested an IoT system that uses meteorological and pollution sensors to collect data and transmit them for evaluation and prediction using neural networks (Bayesian Regularization). They used the past 24-h data to predict the next 24-h period. The study did not use any clear evaluation metric; instead, they presented a comparison of prediction values vs. actual value using different sample sets. Their proposed system uses many sensors to ensure accuracy and minimize monitoring cost. The system is scalable and suitable for big data analysis.

A comprehensive analysis was conducted by [29] to study design considerations and development for air pollution monitoring using the IoT paradigm and edge computing. They calibrated data collected from sensors using Arduino as an edge computing device before further processing. The Air Quality Index (AQI) was calculated at the edge device and was not sent to the cloud unless it was above a specific limit. Data are collected in an IBM cloud for visualization and further processing. They calculated outdoor AQI using the three dominant pollutants (PM_2.5_, PM_10_ and CO_2_). The evaluation was done by calculating AQI and comparing a setup where measurements were flattened, calibration and accumulation algorithms were employed, and another setup where measurements were raw. They developed a system that saves bandwidth and energy consumption. However, further processing by the edge can save even more bandwidth and energy consumption.

A system that can be applied to monitor pollution levels of a smart city is proposed in [30]. It is used primarily for monitoring rather than conducting prediction of future pollution levels. Its primary focus is the security of the data. Besides, it tackles security issues of that kind of IoT system. There is no evaluation metric of their system, only a proof of concept. Their IoT solution is scalable, reliable, secure and has HA (high availability). However, it relies on central management and central prediction rather than performing prediction on edge devices.

In [31], the authors proposed a prediction model that uses data from IoT sensors deployed across a smart city. This model uses LSTM to predict O_3_ and NO_2_ pollution levels, then it calculates AQI and classify the output as an alarm-level of (Red, Yellow and Green). They used RMSE and MAE to evaluate the prediction performance, whereas F1-score was used to evaluate classification accuracy. LSTM was compared to SVR as a baseline, and LSTM was proven to be a better algorithm. However, their research did not include a comparison to other works and used only one base model.

Related work can be summarized in Table 1.

**Table 1:** Related work summary

Reference	Evaluation metrics	Pros	Cons
[20]	MAE, RMSE, IA	Feasibility and practicality were verified experimentally for forecasting PM_2.5_ using their proposal	Algorithmic predictions did not follow real trend accurately and were a bit shifted and disordered
[22]	MAE, RMSE	CNN-GRU and CNN-LSTM worked better for PM_10_ and PM_2.5_, respectively	Hybrid models weakly predicted future highest and lowest levels of PM_2.5_
[23]	RMSE, R^2^	After using multiple algorithms, it was found that Extra Trees gives the best performance	The study was limited in the number of machine learning algorithms compared. There was a bit of a shift between actual and predicted values for most algorithms
[24]	RMSE, MAE	CNN could extract air quality features, shortening training time, whereas LSTM could perform prediction using long-term historical input data	More evaluation parameters, stating closeness to real values like R^2^ or IA rather than only errors metrics, could have been used to confirm their models' performance
[25]	NMSE, FB and FA2	PM_2.5_ concentration was predicted using meteorological parameters and PM_10_ and CO without a history of PM_2.5_ itself	More machine learning models could have been used to test their methodology further
[26]	SMAPE	The ensemble of the three models (AccuAir) proved to be better than the individual components tested	They did not use LSTM in their Seq2Seq model, although it was proven to be very efficient in time series prediction
[27]	RMSE, MAE and MAPE	Their model was compared to Multilayer Perceptron (MLP) and LSTM models and proved to be more stable and accurate	Their system predicts only the daily average and cannot be deployed to predict the hourly or real-time concentration of PM_2.5_
[28]	A comparison of prediction values vs. real value using different sample sets	Their proposed system uses many sensors to ensure accuracy and minimize monitoring cost. The system is scalable and suitable for big data analysis	The study did not use any clear evaluation metric; instead, they presented a comparison of prediction values vs. actual value using different sample sets
[29]	Calculating AQI and comparing two setups with and without measurements flattened and calibration and accumulation algorithms employed	They developed a system that saves bandwidth and energy consumption	Further processing by the edge can save even more bandwidth and energy consumption. However, no prediction exists on the edge devices or the cloud side
[30]	There is no evaluation metric of their system, only a proof of concept	It tackles security issues of that kind of IoT system. Their IoT solution is scalable, reliable, secure and has HA (high availability)	The system is used primarily for monitoring rather than conducting prediction of future pollution levels. It relies on central management and central prediction rather than performing prediction on edge devices
[31]	RMSE, MAE and F1	It comprised both prediction and classification to make an alarm system. LSTM was compared to SVR as a baseline, and LSTM was proven to be a better algorithm	Their research did not include a comparison to other works and used only one base model

## Proposed IoT-based air quality monitoring and prediction system description

**Fig. 1:**
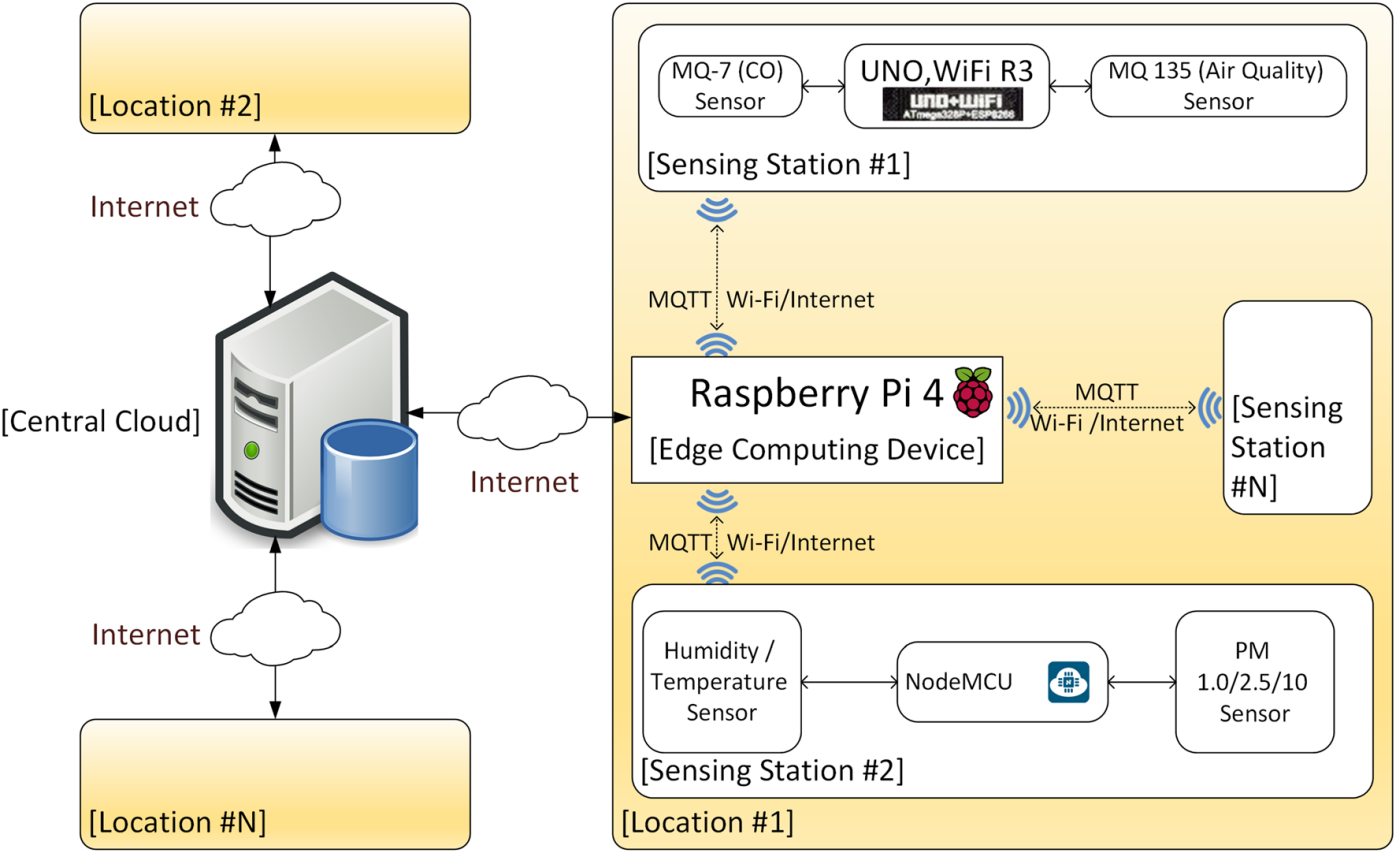
Proposed IoT System architecture

This section proposes a new system that leverages the ever-growing set of single board computers (SBC) that contain hardware powerful enough to perform a reasonable level of computation with low cost and power consumption. The following diagram illustrates the components of the proposed design (Fig. 1).

On the edge of the system exists an instance of SBC, a Raspberry Pi 4. The Raspberry Pi 4 is responsible for controlling and collecting data from multiple sensing stations via Message Queuing Telemetry Transport (MQTT). Hence, the edge device will act as an MQTT broker for all sensing stations, MQTT clients. Each station gathers readings from the connected sensors via a multitude of inputs available in an Arduino-compatible device equipped with Wi-Fi capabilities, such as NodeMCU, Arduino Uno Wi-Fi, Uno, Wi-Fi R3, amongst others. Data could be sent to the Raspberry Pi through its General-Purpose Input Output (GPIO) pins or other inputs if Wi-Fi is unavailable. Sensors may include MQ gas sensors, humidity and temperature sensors like DHT-11 or DHT-22 and PM sensors. The stations may be placed in the same city in industrial or residential locations or distributed across the country, according to the authority’s needs.

After collecting data from the attached sensors for its configured period (mostly 24 h to 1 week) [20, 22], the edge device is responsible for calculating Air Quality Index (AQI) as well as predicting the next time step or steps (minutes, hours, days, real time) according to its configuration. It may also warn its local vicinity or perform other tasks as configured by the authority or its operator. Afterwards, it may compress available readings and send them to the central cloud for further processing and prediction on a large scale. A system composed of these edge devices would broadcast their raw data to the cloud, which helps in making pivotal decisions and predicting next time-steps for the whole area monitored by the system. The cloud would also help estimate and predict AQI for areas without edge devices, and it may even send corrective data to the edge devices to better predict air pollution concentration level in their local region according to data collected from other neighbouring areas.

This system could be used in multiple configurations, including industrial establishments, especially those dealing with environmentally hazardous substances and other factories in general. In addition, the average consumer would benefit from such a system that could work independently from the cloud if required. Also, in governmental settings, this would give the big picture of the air quality situation nationwide. Finally, this system has a flexible configuration as it does not require fixed/static installations and can be mounted on moving vehicles with appropriate adjustments. The system has not been fully implemented yet just the edge part was implemented using a Raspberry Pi 4 device, and the next phase of this research study is to complete the full implementation.

### Practical implications for implementing the proposed system

**Fig. 2:**
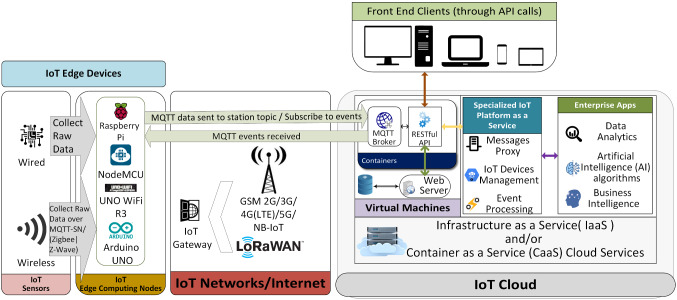
Proposed system data flow architecture

The proposed system will have multiple layers in terms of data flow, as shown in Fig. 2.

The layers presented in the figure above show the logical flow of transmission and processing of data by many devices and networks according to the available resources upon implementation.

The components of the system are:

1. IoT edge devices:
  - The IoT Sensors layer contains the sensors required in the prediction process. The sampling rate can be fixed or controlled by the IoT Edge Computing Nodes layer. This layer can get multiple readings including but not limited to: relative humidity level (%), temperature (°C), altitude (m), pressure (hPa), carbon monoxide CO (ppm), carbon dioxide CO_2_ (ppm), particulate matter of 0.3 ~ 10 µm in diameter (µg/m^3^), ammonium NH4 (ppm), methane CH4 (ppm), wind direction (°deg), wind speed (m/s), detected Wi-Fi networks, and their signal strength in decibels. IoT Edge devices layer comprises:
    i. Wired sensors transmit data through numerous methods, such as (Inter-Integrated Circuits—I2C, Serial Peripheral Interface—SPI, and Universal Asynchronous Receiver/Transmitter—UART) to the next layer.
    ii. Wireless sensors in which data are sent via wireless protocols (ZigBee/Z-Wave). Data could be carried using MQTT over Zigbee protocol called (MQTT-SN).
  - IoT edge computing nodes:
    Here smart edge devices can be used to process collected data and send either a summary or a stream of the current readings to the cloud or perform the required local prediction directly using the computing power available to them. Example of these nodes is SBCs, Arduinos, and Arduino-compatible devices.
2. IoT network/internet:
  Communication between IoT Edge Devices and the IoT cloud is carried through this layer. First, IoT gateways coordinate between various IoT Edge nodes in terms of network usage and cooperation. For example, SBCs from the previous layer could be used as IoT gateways. Then, the connections are relayed to the cloud via many possible network facilities, such as mobile technologies (2G-3G-4G-5G-Narrowband IoT), Low-Power Wide Area Network (LPWAN) technologies including (Long-Range Wide Area Network (LoRaWAN) and Sigfox) or Wi-Fi. Finally, it is required to provide secure and reliable linkage to the IoT Cloud layer with good coverage across the area to be monitored.
3. IoT cloud:
  All data collected from various stations in the system are processed in this part of the data flow. This part could be optional if the prediction is entirely made on the edge devices. However, for a bigger picture and more accurate results, central management and processing add higher value.
  Usually, the processing cloud comprises Infrastructure-as-a-Service (IaaS) or Container-as-a-Service (CaaS) cloud services, on top of which other services may run. For example, MQTT brokers may run in a container hosted in a virtual machine, or they can run directly on the hypervisor if supported like vSphere 7.0 by VMWare [32]. The container can also be run in various systems such as Amazon Elastic Compute Cloud (EC2), serving containers like Docker and Kubernetes. A virtual machine could have a container instance running the MQTT broker and another running web services conforming to REpresentational State Transfer (REST) standards—also known as RESTful web services. Besides, a (not only SQL—NoSQL) database server and a webserver would be in the virtual machine to serve the RESTful requests forwarded by the broker and store data required, respectively. Many virtual machines may exist for multiple areas for scalability. The data stored can be processed, and coordination between IoT devices can be made by a specialized IoT platform as a service software tool. To make large-scale predictions and decisions, data analytics and business intelligence, as well as specialized AI prediction algorithms, may be deployed.
4. Front end clients:
  Web services API calls may be made to deliver helpful information for various clients, create alerts and historical or live maps of the requested area’s situation.

### Prediction algorithms

To help build the proposed system, multiple prediction algorithms were compared to determine the best and most efficient one for use at both the edge and the central cloud.

### Non-linear AutoRegression with eXogenous input (NARX) model

**Fig. 3:**
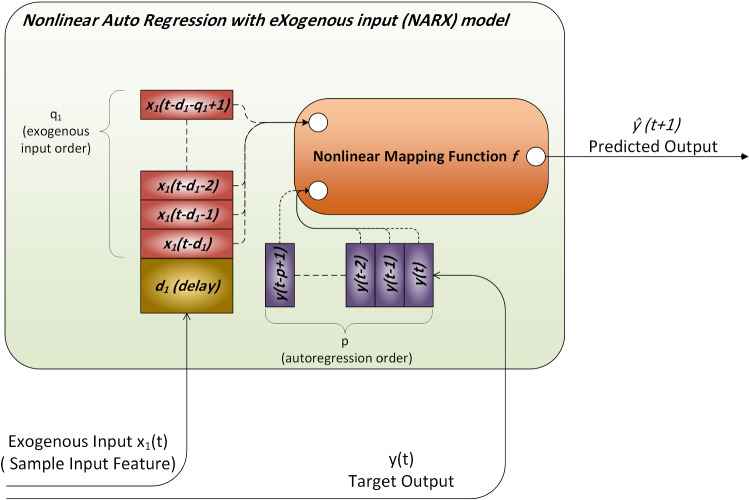
NARX model

NARX is mainly used in time series modelling. It is the non-linear variant of the autoregressive model having exogenous (external) input. The autoregressive model determines output depending linearly on its past values. Hence, NARX relates the current value of a time series to previous values of the same series and current and earlier values of the driving (exogenous) series. A function exists to map input values to an output value. This mapping is usually non-linear—hence NARX—and it can be any possible mapping functions including Neural Networks, Gaussian Processes, Machine Learning algorithms and others. The general concept of NARX is illustrated in Fig. 3 [33].

The model works by inserting input features from sequential time-steps *t* and grouping past time-steps in parallel into the exogenous input order each of length *q*. If required, each of these features can be delayed by *d* time-steps. This means that for each input feature you can choose how many timesteps to include using exogenous order *q* and delay that amount of data by *d*. Figure 3 shows that by including only one input feature marked as *x*_1_ and using *q*_1_ input order and *d*_1_ delay. Meanwhile, the target values are stacked similarly, representing autoregression order of length *p*. Direct AutoRegression (DAR) is another variant in which the predicted output is used as an autoregression source rather than externally [34]. A library named fireTS has been implemented in Python by [35] to apply NARX using any scikit-learn [36] compatible regression library as a mapping function. NARX can be represented mathematically as [34]

1 $$ \hat{y}\left( {t + 1} \right) = f\left( {y\left( t \right), y\left( {t - 1} \right), y\left( {t - 2} \right), \cdots ,y\left( {t - p + 1} \right),X\left( {t - d} \right),X\left( {t - d - 1} \right), X\left( {t - d - 2} \right), \cdots , X\left( {t - d - q + 1} \right)} \right) $$

where $\widehat{y}$ is the predicted value, $f\left(.\right)$ is the non-linear mapping function,$y$ is the target output at various time-steps $t$, $p$ is the order of target outputs (autoregression) used specifying how many time-steps to use of the target of prediction, $X$ is input features matrix, $q$ is a vector specifying the order of exogenous input determining how many time-steps to inject from each of the input features, and $d$ is a vector representing the delay introduced to each of the input features.

### Long short-term memory (LSTM)

Long short-term memory algorithm is one of the algorithms that are used frequently for analysing time series data. It receives not only the present input but results from the past as well. This process is executed by utilizing the output at time (*t*-1) to be the input at time (*t*), accompanied by the fresh input at time (*t*) [37]. Hence, there is' memory' stored within the network, in contrast to the “feedforward networks”. This approach is a crucial feature of LSTM as there exists constant information about the preceding sequence itself and not just the outputs [38]. Air pollutants vary over time and health threats are related to long-term exposures to PM_2.5_. During long periods, it is manifest that the best forthcoming air pollution predictor is the prior air pollution [39]. Simple Recurrent Neural Networks (RNNs) often require finding links among the final output and input data. Storing several time-steps before are limited as there exist several multiplications (an exponential number) that occur within the net hidden layers. These multiplications result in derivatives that will progressively fade away; consequently, the computation process to execute a learning task becomes difficult for computers and networks [37].

**Fig. 4:**
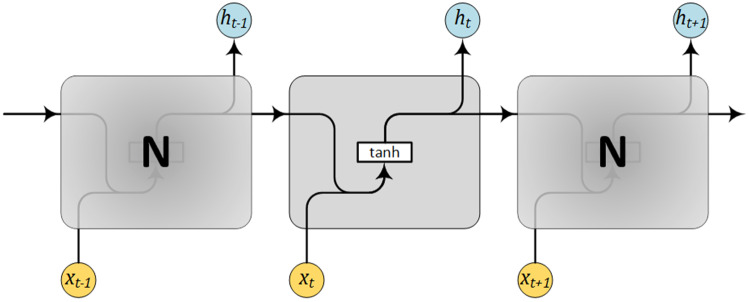
Simple RNN with one layer and no gated memory cells

**Fig. 5:**
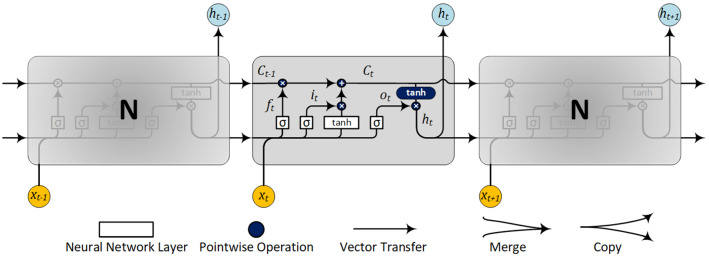
LSTM RNN elemental network structure

For this reason, LSTM is a suitable model because it preserves errors within a gated cell. On the other hand, simple RNN usually has low accuracy and major computational bottlenecks. A comparison between simple RNN and LSTM RNN is presented in Figs. 4, 5 [40].

It is evident from Figs. 4, 5 that the memory elements in Fig. 5 are the main difference between the structure of RNN and LSTM.

The process of forward training of LSTM is formulated via the following equations [41]:

2 $$ f_{t} = \sigma \left( {W_{f} \cdot \left[ {h_{t - 1} ,x_{t} } \right] + b_{f} } \right) $$

3 $$ i_{t} = \sigma \left( {W_{i} \cdot \left[ {h_{t - 1} ,x_{t} } \right] + b_{i} } \right) $$

4 $$ C_{t} = f_{t} *C_{t - 1} + i_{t} *\tanh \left( {W_{C} \cdot \left[ {h_{t - 1} ,x_{t} } \right] + b_{c} } \right) $$

5 $$ o_{t} = \sigma \left( {W_{o} \cdot \left[ {h_{t - 1} ,x_{t} } \right] + b_{o} } \right) $$

6 $$ h_{t} = o_{t} *\tanh \left( {C_{t} } \right) $$

where ${i}_{t}$,${o}_{t}$ and ${f}_{t}$ are activation of the input gate, output gate and forget gate, respectively; ${C}_{t}$ and ${h}_{t}$ are the activation vectors for each cell and memory block, respectively; and $W$ and $b$ are the weight matrix and bias vector, respectively. Also $\sigma \left(\bullet \right)$ is considered the sigmoid function defined in (7) and $tanh\left(\bullet \right)$ is the tanh function, specified in (8).

7 $$ \sigma \left( x \right) = \frac{1}{{1 + e^{ - x} }} $$

8 $$ \tanh \left( x \right) = \frac{{e^{x} - e^{ - x} }}{{e^{x} + e^{ - x} }} $$

### Random forests (RF)

The algorithm of Random forests can be defined as a collection of decision trees, where every single tree is employing the best split for its construction. Each node in the predictor’s subset is picked randomly at that node. Then, for the prediction step, the majority vote is taken.

Random forests possess two parameters:

- *m*_try_: number of predictors sampled for the splitting step at every node.
- *n*_tree_: number of grown trees.

Random Forest algorithm starts by first obtaining *n*_tree_ bootstrap samples from the original data. Next, an unpruned classification or regression tree is grown using mtry of sampled random predictors for each sample. Then, the fittest split is chosen at each node. Eventually, predictions are carried out using the predictions aggregation of *n*_tree_ trees, such as the average or median, for regression and majority poll for classification.

To calculate the error rate, predictions of the out-of-bag samples, which means the data are not included in a bootstrap sample, are used [42, 43].

### Extra trees (ET)

Extra Trees machine learning algorithm depicts a tree-based ensemble approach operated in supervised regression and classification problems. Its central notion is about constructing regression trees ensemble or unpruned decision trees per the top-down classical procedure. Moreover, it builds wholly randomized trees whose structures are separate from the learning sample result values in extreme cases.

Extra Trees and Random Forest revolve around the same idea. In addition, though, Extra Trees selects the best feature at random in conjunction with the corresponding value during splitting the node [44]. Another distinction between Extra Trees and Random Forest is that Extra Trees uses all components of the training dataset to train every single regression tree, whereas Random Forest trains the model using the bootstrap replica technique [45].

### Gradient boost (GB)

Gradient Boost is one of the ensemble-learning techniques in which a collection of predictors come together to give a final prediction. Boosting requires predictors to be made sequentially; hence training data are fed into the predictors without replacement leading to new predictors learning from previous predictors [46]. This sequential process reduces the time required to reach actual predictions. In addition, gradient boosting uses weak learners/predictors to build a more complex model additively. These predictors are usually decision trees.

### Extreme gradient boost (XGB)

XGBoost is another ensemble scalable machine learning algorithm for gradient tree boosting used widely in computer vision, data mining, and other domains [47]. The ensemble model used in XGBoost—usually a tree model — is trained additively until stopping criteria are satisfied, such as early stopping rounds, boosting iterations count, amongst others. The objective is to optimize the $t$-th iteration by minimizing the subsequent approximated formula [47]:

9 $$ {\mathcal{L}}^{\left( t \right)} \simeq \mathop \sum \limits_{i = 1}^{n} \left[ {l\left( {y_{i} ,\hat{y}_{i}^{{\left( {t - 1} \right)}} } \right) + \partial_{{\hat{y}^{{\left( {t - 1} \right)}} }} l\left( {y_{i} ,\hat{y}_{i}^{{\left( {t - 1} \right)}} } \right)f_{t} \left( {x_{i} } \right) + \frac{1}{2}\partial_{{\hat{y}^{{\left( {t - 1} \right)}} }}^{2} l\left( {y_{i} ,\hat{y}_{i}^{{\left( {t - 1} \right)}} } \right)f_{t}^{2} \left( {x_{i} } \right)} \right] + \Omega \left( {f_{t} } \right) $$

where ${\mathcal{L}}^{\left(t\right)}$ is the solvable objective function at the $t$-th iteration, $l$ is a loss function that calculates the difference between the prediction $\widehat{y}$ of the $i$-th item at the $t$-th iteration and the target ${y}_{i}$, ${\partial }_{{\widehat{y}}^{\left(t-1\right)}}l\left({y}_{i},{\widehat{y}}_{i}^{\left(t-1\right)}\right)$ is first-order gradient statistics on the loss function and ${\partial }_{{\widehat{y}}^{\left(t-1\right)}}^{2}l\left({y}_{i},{\widehat{y}}_{i}^{\left(t-1\right)}\right)$ is the second-order, ${f}_{t}\left({x}_{i}\right)$ is the increment.

XGBoost is currently one of the most efficient open-source libraries, as it allows for fast model exploration and uses minimal computing resources. These merits led to its use as a large-scale, distributed, and parallel solution in machine learning. Besides, XGBoost generates feature significance scores according to feature frequency use in splitting data or based on the average gain a feature introduces when used during node splitting across all trees formed. That characteristic is of great use and importance for analysing factors that increase PM_2.5_ concentrations.

### Random forests in XGBoost (XGBRF)

Gradient-boosted decision trees and other gradient-boosted models can be trained using either XGBoost or Random Forests. This training is possible because they have the exact model representation and inference, but their training algorithms are different. XGBoost can use Random Forests as a base model for gradient boosting or can be used to train standalone Random Forests. In XGBRF training, standalone random forest is the focus. This algorithm is a Scikit-Learn wrapper introduced in the open-source library of XGBoost, and it is still experimental [48]; this means that the interface can be updated anytime.

### Proposed NARX hybrid architecture

**Fig. 6:**
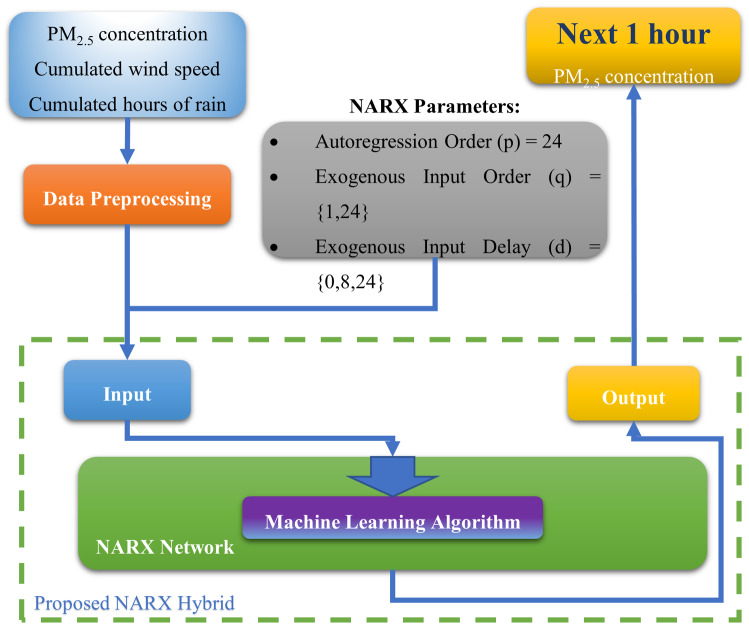
Proposed architecture diagram

Our proposed architecture uses NARX’s non-linear mapping function as a host for machine learning algorithms. As Fig. 6 illustrates, the input features are passed through the pre-processing process, which removes invalid data and normalizes features and converts categorical features to numeric values. Data are then split into training and testing segments. The training segment is the first four years of data, and the testing uses the last year of the dataset described in section “Data Description and Preprocessing”. Then NARX trains the machine learning (ML) algorithm with data in each epoch as defined by its parameters. The system is then evaluated using the fifth-year test data.

The proposed architecture can be described in the following steps:
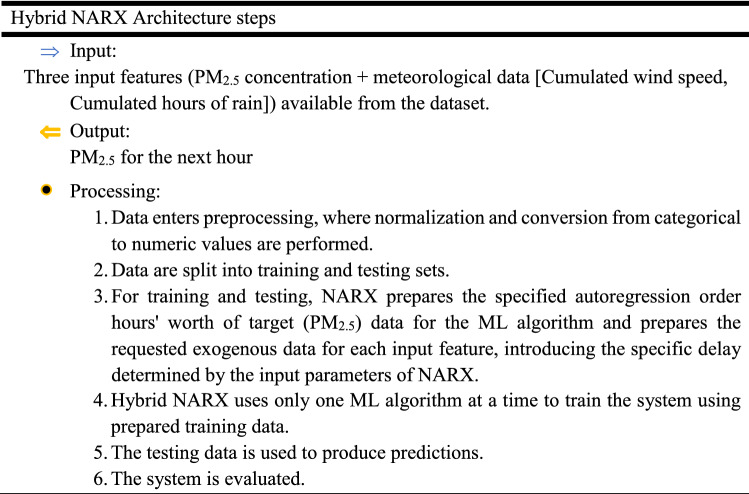


### Performance evaluation

#### Evaluation metrics

To assess the performance of the prediction model used and reveal any potential correlation between the predicted and actual values, the following metrics are used in our experiments.

#### Root mean square error (RMSE)

Root mean square error computes the square root of the mean for the square of the differences between predicted and actual values. It is computed as [49]:

10 $$ {\text{RMSE}} = \sqrt {\frac{{\mathop \sum \nolimits_{i = 1}^{n} \left( {P_{i} - A_{i} } \right)^{2} }}{n}} $$

where *n* is the number of samples, ${P}_{i}$ and ${A}_{i}$ are the predicted and actual values, respectively.

RMSE has the same measurement unit of the predicted or actual values, which is in our study μg/m^3^. The less RMSE value is the better the model prediction performance.

#### Normalized root mean square error (NRMSE)

Normalizing root mean square error has many forms. One form is to divide RMSE by the difference between maximum and minimum values in the actual data. Comparison between models or datasets with different scales is better performed using NRMSE. The equation used for its calculation is[50]:

11 $$ {\text{NRMSE}} = \frac{{{\text{RMSE}}}}{{{\text{Max}}\left( {A_{i} } \right) - {\text{Min}}\left( {A_{i} } \right)}} $$

#### *Coefficient of determination (R*^*2*^*)*

This parameter evaluates the association between actual and predicted values. It is determined as [51]:

12 $$ R^{2} = 1 - \frac{{\mathop \sum \nolimits_{i = 1}^{n} \left( {A_{i} - P_{i} } \right)^{2} }}{{\mathop \sum \nolimits_{i = 1}^{n} \left( {A_{i} - \overline{A}} \right)^{2} }} $$

where *n* is the records count, ${P}_{i}$ and ${A}_{i}$ are the predicted and actual values, respectively. $\overline{A }$ represents the mean measured value of the pollutant.

As for the unit of measurement, ${R}^{2}$ is a descriptive statistical index. Hence, it has no dimensions or unit of measurement. If the prediction is completely matching the actual value, then ${R}^{2}=1$. A baseline model where the predicted value is always equal to the mean actual value will produce ${R}^{2}=0$. If predictions are worse than the baseline model, then ${R}^{2}$ will be negative.

#### Index of agreement (IA)

A standardized measure of the degree of model forecasting error varying between 0 and 1; proposed by [52]. This measure is described by:

13 $$ {\text{IA}} = 1 - \frac{{\mathop \sum \nolimits_{i = 1}^{n} \left( {\left| {P_{i} - A_{i} } \right|} \right)^{2} }}{{\mathop \sum \nolimits_{i = 1}^{n} \left( {\left| {P_{i} - \overline{A}} \right| + \left| {A_{i} - \overline{A}} \right|} \right)^{2} }} $$

where *n* is the samples count, ${P}_{i}$ and ${A}_{i}$ are the predicted and actual measurements, respectively. $\overline{P }$ and $\overline{A }$ represent the mean of predicted and measured value of the target, respectively. It is a dimensionless measure where 1 indicates a complete agreement and 0 indicates no agreement at all. It can detect proportional and additive differences in the observed and predicted means and variances; however, it is overly sensitive to extreme values due to the squared differences.

### Data description and preprocessing

The dataset used was acquired from meteorological and air pollution data from 2010 to 2014 [21] for Beijing—China, published as a dataset in the University of California, Irvine (UCI) machine learning repository. This dataset was employed just for evaluation purposes, and in the following research, data from Egypt will be used when available from authoritative air pollution stations. The dataset encompasses hourly information about numerous weather conditions, such as (dew point, temperature) °C, (pressure) hPa, (combined wind direction, cumulated wind speed) m/s, cumulated hours of rain and cumulated hours of snow. It also includes PM_2.5_ concentration in µg/m^3^. Only cumulated wind speed and cumulated hours of rain, as well as PM_2.5_, were used in our experiments. All records missing PM_2.5_ measurements were removed.

Before being used in the chosen prediction algorithms, the dataset was converted into a time series dataset to solve a supervised learning problem [53]. To predict PM_2.5_ of the next hour, data from the earlier 24 h were used. The transformation was performed via shifting records up by 24 positions (the hours employed as the basis for prediction). Then these records were placed as columns next to the present dataset, and this process was repeated recursively to get this form; dataset (*t*-*n*), dataset (*t*-*n*-1), …, dataset (*t*-1), dataset (*t*). This shifting was used in algorithms that were used independently from NARX hybrid architecture. To evaluate the algorithms properly, K-Fold = 10 splitting method was used. K-Fold splits the dataset records into *n* sets using *n*-1 as training and one set as the test in a rotating manner. No randomization or shuffling was used with K-Fold splitting. The input for the LSTM algorithm was rescaled using scikit-learn StandardScaler API [54] using default parameters. Standard Scaler removes the mean and scales to unit variance. To ensure no data leakage [56], scaling and inverse scaling for training set and test set were done separately. Dataset statistics are displayed in Table 2.

**Table 2:** Dataset statistics

	Cumulated wind speed	Cumulated hours of rain	PM_2.5_
Count	43,824	43,824	41,757
Mean	23.88914	0.194916	98.61321
Standard deviation	50.01006	1.415851	92.04928
Minimum	0.45	0	0
Percentile (25%)	1.79	0	29
Percentile (50%)	5.37	0	72
Percentile (75%)	21.91	0	137
Maximum	585.6	36	994
Empty count	0	0	2067
Loss percentage	0.00%	0.00%	4.95%
Coverage percentage	100.00%	100.00%	95.28%

## Results analysis and discussion

Experiments were run on two platforms for validation purposes; on an edge device and a PC. The PC had an Intel processor Core i7 6700 @3.4 GHz quad-core with hyper-threading enabled alongside 16 GB of DDR4 RAM. The edge device was a Raspberry Pi 4 Model B (referred to afterwards as RP4) with 4 GB LPDDR4-3200 SDRAM. The devices were dedicated only to run the experiments with no other workloads. As stated, the input was shifted by 24 h to adapt for a time series prediction, but only for the algorithms that were not used as base models in NARX hybrid methods. The same shifted input was supplied to six methods: LSTM, RF, ET, GB, XGB, and XGBRF.

The proposed NARX hybrid architecture hosted six machine learning algorithms, LSTM, RF, ET, GB, XGB, and XGBRF.

As for algorithms parameters, LSTM had three layers (1) an input layer of 128 nodes, (2) a hidden layer of 50 nodes, and (3) an output layer of one node. LSTM was executed using a batch size of 72 and 25 epochs and used Rectified Linear Unit (ReLU) activation function as well as Adaptive Moment Estimation (Adam) optimizer to minimize the loss function (MAE). This configuration was used in [23]. All other algorithms used default values as indicated by scikit-learn API [54]. NARX used parameters of 24 for auto-order of PM_2.5_ and four combinations of exogenous delay (ed) and exogenous order (eo) for the combined wind speed and cumulated hours of rain for each hosted algorithm namely ([00, 00], [01, 01]), ([00, 00], [24, 24]), ([08, 08], [01, 01]), and ([24, 24], [24, 24]), respectively.

All methods were executed in parallel on all Central Processing Unit (CPU) cores to boost performance. The following figures show 48-h-sample time-steps predicted via our tests versus real values for one of the ten runs performed.

Figures 7, 8, 9, 10, 11, 12, 13, 14, 15, 16, 17, 18 show the values of predicting two days in one-hour time-steps of the fifth fold of the K-Fold splitting using the algorithms mentioned above accompanied by actual data to assess their performance on both a PC and an RP4. Those figures compare the actual measured data to the predicted value using a specific algorithm without NARX and with various NARX configurations.

**Fig. 7:**
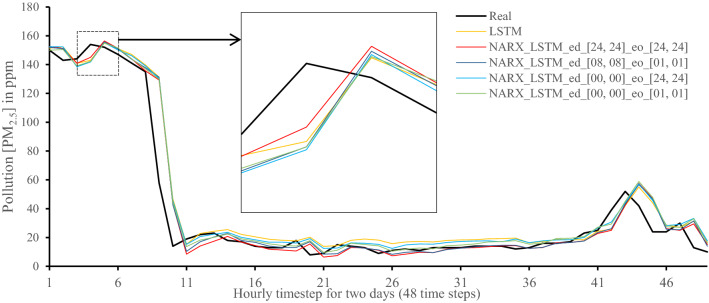
Real vs. Prediction for the proposed NARX hybrid and LSTM run on PC

**Fig. 8:**
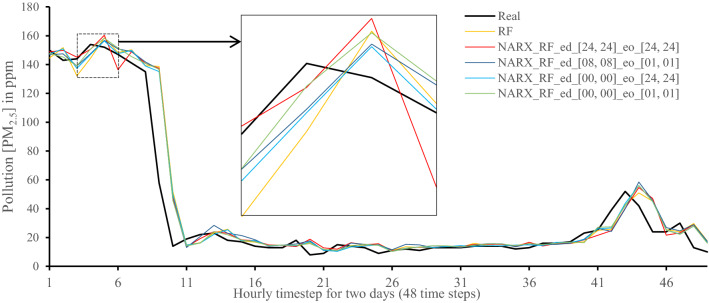
Real vs. Prediction for the proposed NARX hybrid and Random Forests run on PC

**Fig. 9:**
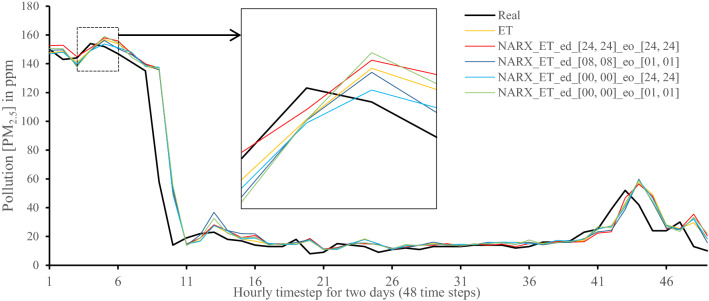
Real vs. Prediction for the proposed NARX hybrid and Extra Trees run on PC

**Fig. 10:**
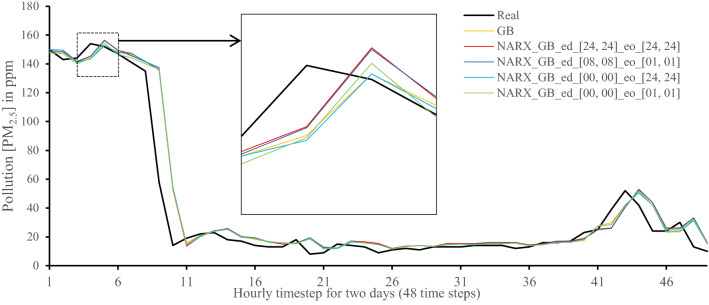
Real vs. Prediction for the proposed NARX hybrid and Gradient Boost run on PC

**Fig. 11:**
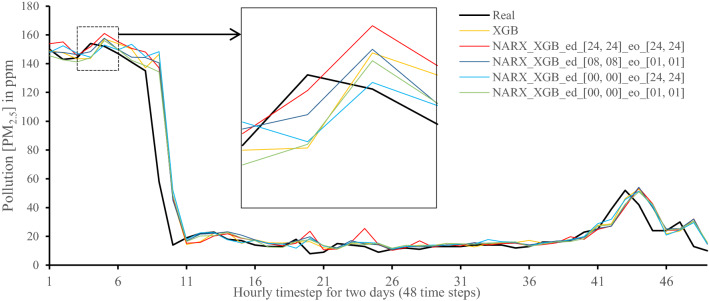
Real vs. Prediction for the proposed NARX hybrid and Extreme Gradient Boost run on PC

**Fig. 12:**
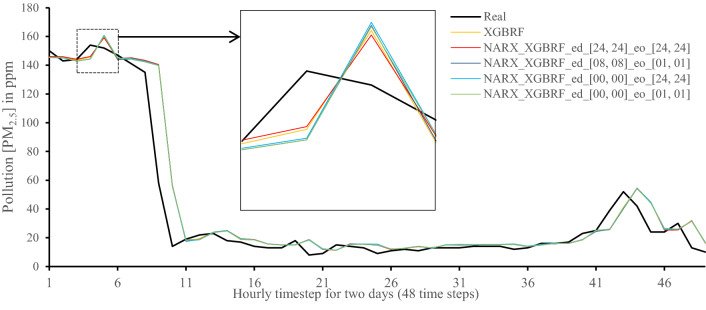
Real vs. Prediction for the proposed NARX hybrid and Random Forests in XGBoost run on PC

**Fig. 13:**
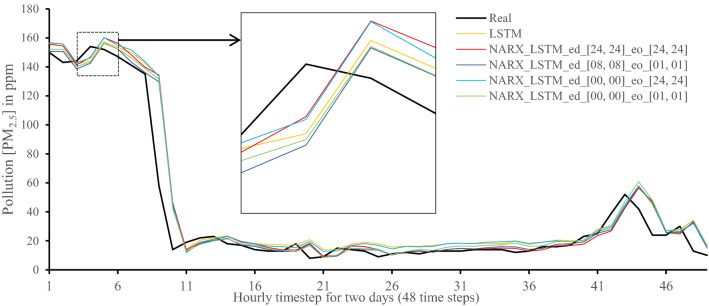
Real vs. Prediction for the proposed NARX hybrid and LSTM run on Raspberry Pi 4

**Fig. 14:**
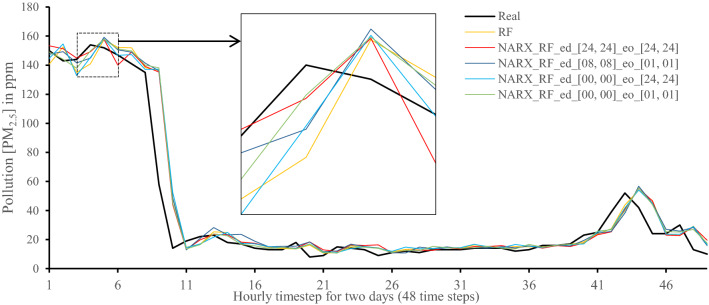
Real vs. Prediction for the proposed NARX hybrid and Random Forests run on Raspberry Pi 4

**Fig. 15:**
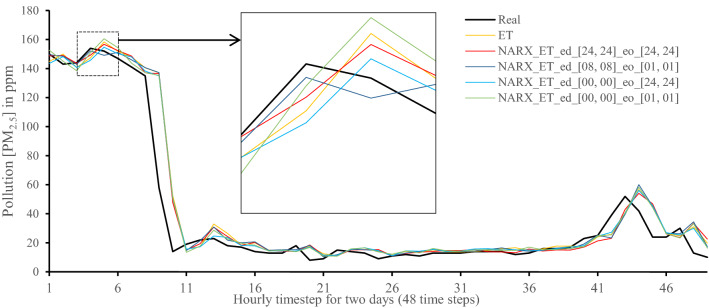
Real vs. Prediction for the proposed NARX hybrid and Extra Trees run on Raspberry Pi 4

**Fig. 16:**
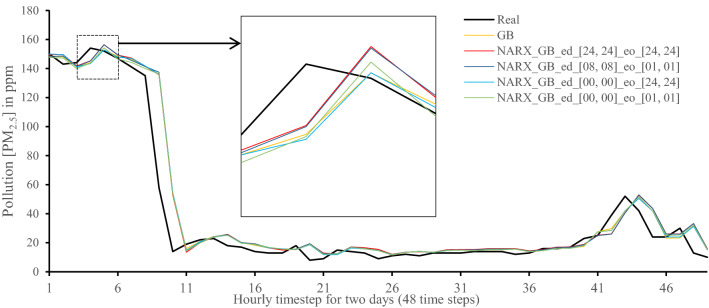
Real vs. Prediction for the proposed NARX hybrid and Gradient Boost run on Raspberry Pi 4

**Fig. 17:**
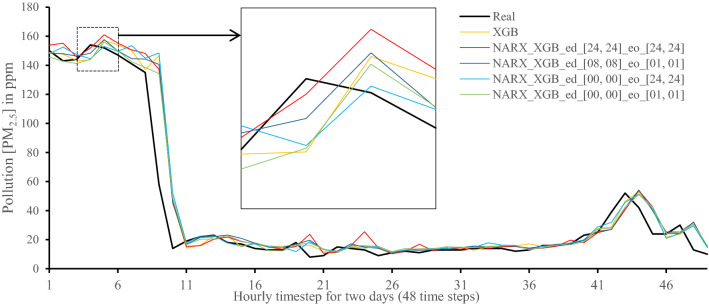
Real vs. Prediction for the proposed NARX hybrid and Extreme Gradient Boost run on Raspberry Pi 4

**Fig. 18:**
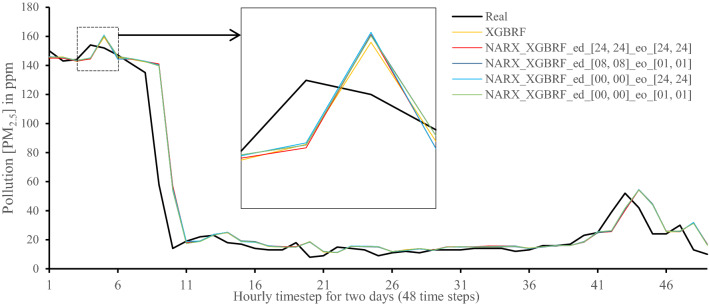
Real vs. Prediction for the proposed NARX hybrid and Random Forests in XGBoost run on Raspberry Pi 4

It is worth mentioning that there is almost always a time-shift in prediction versus real values. Table 3 compares performance metrics for experiments run on the PC as well as the RP4. The arrows next to the evaluation parameter names indicate the direction where better results are, hence the upward arrow indicates that higher values are better results, and the downward arrow indicates that lower values are better results. The best values were coloured in green, while the worst were coloured in red, whereas purple represents the chosen balanced value. The evaluation metrics used were RMSE, NRMSE, *R*^2^, and IA, and training duration in seconds (*T*_tr_)—as measured by Python.

**Table 3:**
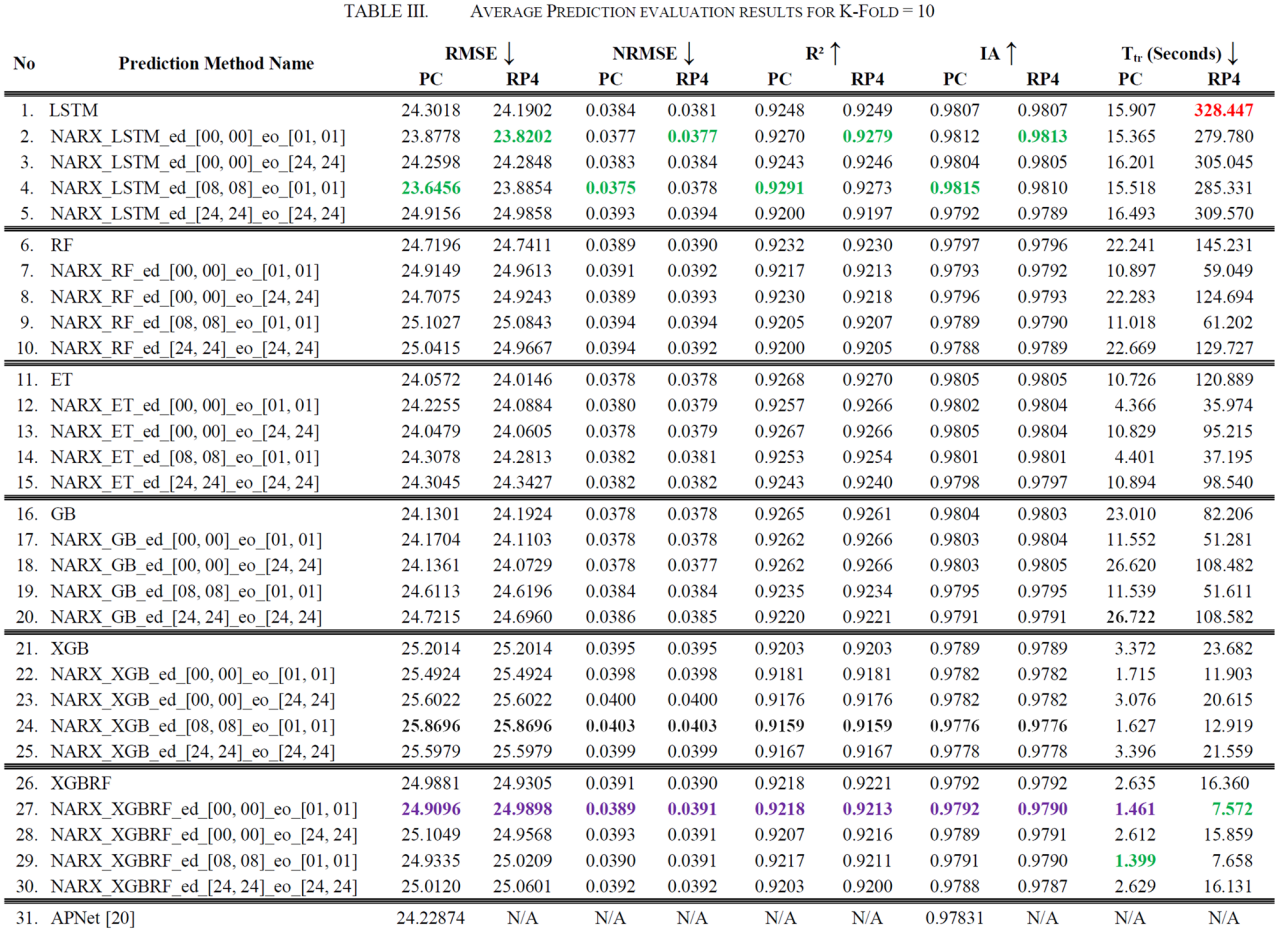
Average Prediction evaluation results for K-Fold = 10

**Fig. 19:**
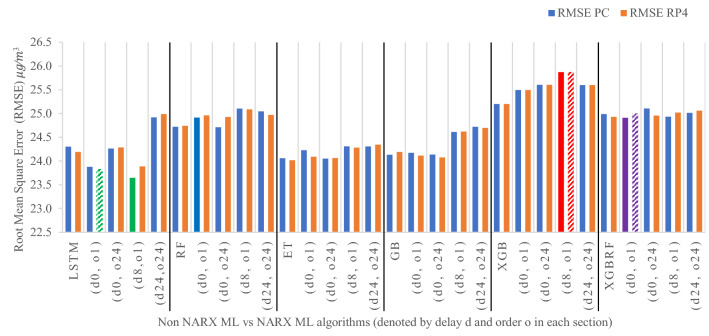
Comparison of the proposed NARX hybrid with other ML algorithms with respect to Root Mean Square Error on PC and Raspberry Pi 4

**Fig. 20:**
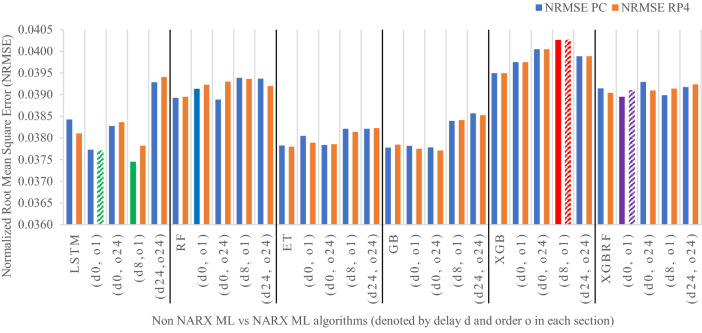
Comparison of the proposed NARX hybrid with other ML algorithms in terms of Normalized Root Mean Square Error on PC and Raspberry Pi 4

**Fig. 21:**
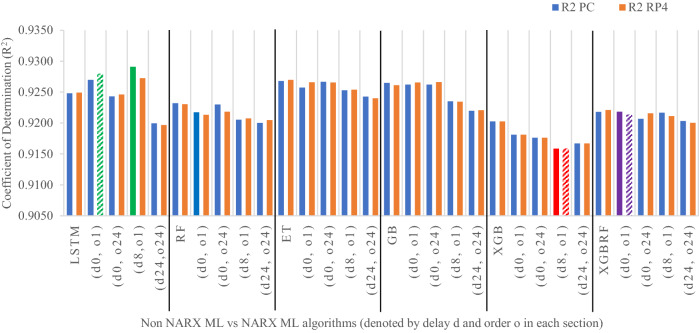
Comparison of the proposed NARX hybrid with other ML algorithms in terms of Coefficient of Determination on PC and Raspberry Pi 4

**Fig. 22:**
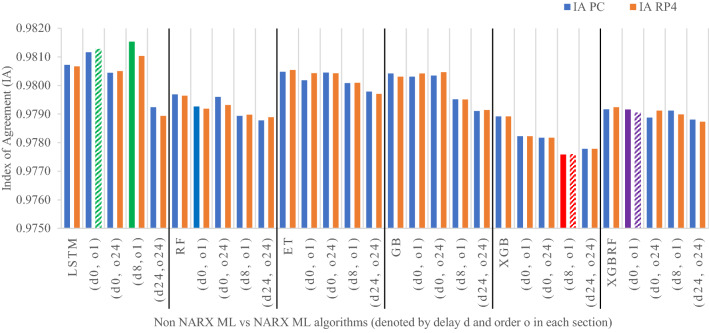
Comparison of the proposed NARX hybrid with other ML algorithms in terms of Index of Agreement on PC and Raspberry Pi 4

**Fig. 23:**
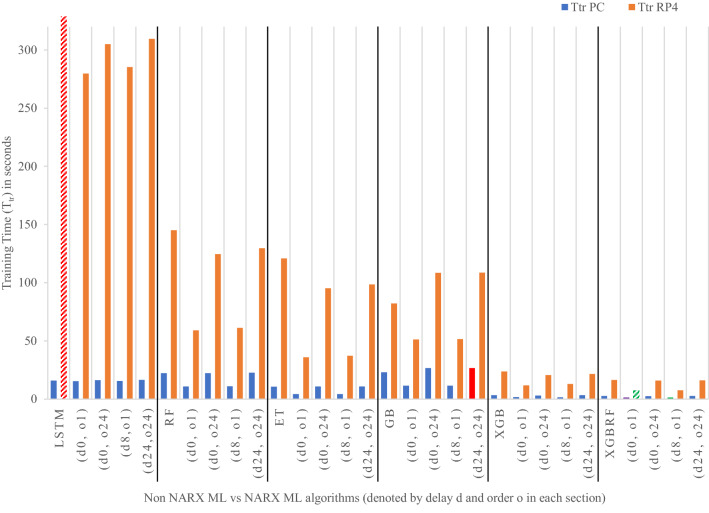
Comparison of the proposed NARX hybrid with other ML algorithms in terms of training duration on PC and Raspberry Pi 4

Figures 19, 20, 21, 22, 23 illustrate these results visually for easier comparison. In all subsequent figures, the worst value bar was coloured in red for the PC and striped red for the RP4. In contrast, the best value bar was coloured in green for the PC and striped green for the RP4. The chosen balance bar was coloured in purple for PC and striped purple for RP4. As the values of exogenous delay and exogenous order for both features are the same in each variation, the name has been shortened in the figures from ed_[xx, xx] and eo_[yy, yy] to be (dx, oy). In addition, each figure is split into sections with each section representing the non-NARX algorithm followed by the NARX variations designated only by (dx, oy) pairs.

In general, all methods examined perform well above 0.9 in R^2^ for both PC and RP4 configurations. The use of NARX allowed showing the effect of exogenous variables on the prediction process of PM_2.5_. Using NARX, the amount of past data for each exogenous (external) variable can be specified as well as how much delay to be introduced for the used data. This delay and exogenous order can indicate the exact effect of the external inputs on the target of predictions. Also, the delay between the external input and the target for prediction (exogenous delay—ed) can be sourced from the physical relation between those external inputs and the target. For example, an increase in the wind speed could help predict pollution level not in the exact near future but after a delay of several hours. In addition, the extended history of a particular external input could mislead the prediction of the target pollutant.

As the results show, the usage of less external data in LSTM (i.e., the exogenous order—eo—of NARX) led to better prediction in general. Nevertheless, in Random Forest and Extra Trees, more external variables data led to better results. This improvement is evident from Figs. 8, 9 for the PC and Figs. 14, 15 for the RP4 where extra information with no delay is the best fit. This observed behaviour could be due to the fact that LSTM contains memory cells that perform better if fewer external data are fed into the training process, creating more focus on the predicted target. This effect can be seen on both Figs. 7, 13 in the zoomed window showing NARX_LSTM_ed_[08, 08]_eo_[01, 01] and NARX_LSTM_ed_[00, 00]_eo_[01, 01], respectively, to be closer to the real values than other methods. In fact, the averaged results of K-Fold = 10 indicate that NARX_LSTM_ed_[08, 08]_eo_[01, 01] is the best algorithm when executed on a PC while NARX_LSTM_ed_[00, 00]_eo_[01, 01] is the best algorithm when executed on a RP4. Due to randomness in LSTM, there is a little difference between results obtained from the devices used, but the consistency of the results is better when using lower exogenous input orders, which proves that LSTM performs better with less interference from exogenous inputs. In addition, less exogenous inputs mean fewer data used for training and hence less training time.

It can be noted that Extreme Gradient Boost (XGB) has no randomness in its processing, leading to typical results being produced across the PC and the RP4 with or without NARX. Nonetheless, XGB scores the lowest performance as indicated in Figs. 11, 17 by the irregular pattern in prediction (a few spikes in the rather staple period). Despite this low prediction performance, the speed of XGB is very high. XGBRF surpasses XGB with even better prediction performance. This improvement is apparent in Figs. 12, 18, where prediction is smoother than XGB. In a RP4, it is important to save energy as less time means less computing power needed and faster results. Hence, NARX_XGBRF can be a very good candidate to run as a predictor on edge devices if the deployed system requires a quick prediction. However, if the system deployed does not require quick prediction, then NARX_LSTM is the best performant. Comparing our results to [20], the average of NARX_LSTM_ed_[08, 08]_eo_[01, 01] on a PC outperforms their APNet average in terms of RMSE (23.6456 vs. 24.22874) as well as IA (0.9815 vs. 0.97831).

## Conclusion

This paper proposed and evaluated a hybrid NARX architecture hosting many machine learning algorithms involved in predicting PM_2.5_ concentration in the atmosphere using the previous 24 h data of cumulated wind speed and cumulated hours of rain to predict the next hour. The experiments were conducted on both a regular PC and an SBC, namely Raspberry Pi 4. Besides, an IoT system is proposed to better monitor and predict Air Quality Index (AQI) by combining sensors and SBCs and a central cloud into an edge computing paradigm. The proposed system is flexible and usable in multiple configurations, including industrial, governmental, and household. The use of edge devices to predict air pollution is essential as it allows for quicker response in case of air pollution incidents or the case where connection to the internet is deficient or in an isolated remote site. The performance of the Machine Learning algorithms used in this work was investigated by applying them to the same dataset. In terms of the correlation between actual and predicted results, NARX/LSTM shows the best performance by providing more accurate results than a state-of-the-art deep learning hybrid method named APNet. To be able to run efficiently on edge devices, fast prediction algorithms are preferable. The obtained results indicate that XGB related methods are fast and the best method for both efficiency and accuracy is NARX/XGBRF.

### Future work

There are various directions to be explored after this work. First, the proposed IoT system can be fully implemented and evaluated in terms of delay in various components and prediction performance. Second, the proposed system can be tested for various scenarios and can be optimized by automatically switching the context due to criteria defined by system operators. For example, the system can be switched from taking samples every 8 h in light pollution to taking more samples and giving better prediction if pollution increases. Third, complete exploration of NARX, with more variation of exogenous order and exogenous delay, can also be done. Fourth, having multiple nodes capable of running prediction algorithms paves the way for distributed computing and optimizations of speed and reliability. In addition, optimizing LSTM to run on edge devices is another step to improve prediction performance. This improvement can be made using GPU processing or Google Coral Edge TPU.
